# Veronal Rashes

**Published:** 1908-03-14

**Authors:** 


### VERONAL RASHES.

Invaluable though veronal has proved to be in certain conditions of sleeplessness, the drug has already shown itself capable of producing certain symptoms, some of which, like the delirium it may cause, are serious, whilst others are only important in that an error of diagnosis may readily be made if they cannot be traced to their real cause. Amongst the latter may be mentioned the erythematous, scarlatiniform eruption upon the skin that may result from the use of veronal.

The eruption in this condition is transient, and it seems to do no harm; so that its occurrence appears to be no contra-indication to the use of the drug. The importance of the eruption is chiefly that it may be mistaken for that of scarlet fever if the observer is not alive to the fact that it may be caused by the veronal itself.

Cases of the kind have been reported upon the Continent by Dr. Zengerly and others. The following are two examples:—

A woman aged fifty was suffering from melancholia and extreme sleeplessness. For the latter all sorts of hypnotics had been used, with more or less ill-success, and, finally, veronal was tried in its turn. In fifteen-grain doses it seemed to work well, and it was repeated on consecutive nights for some time. On the day after the tenth dose the patient complained of a burning sensation in the precordial region. Nothing abnormal could be detected at the time either in the skin or in the sounds or action of the heart. That same evening, however, the chest, abdomen, back, and limbs came out in a uniform strawberry-red erythematous rash, whilst the temperature rose to 101° F. at the same time.

The possibility of the presence of scarlet fever immediately suggested itself, and had there been any chance of contagion having reached the patient, or if she had any severe sore throat or any albuminuria, she would almost certainly have been sent to a fever hospital. Even as it was, the clinical picture of the skin was so like that of scarlet fever that the nature of the trouble was open to doubt at the time. The patient, however, could not have been exposed to scarlatinal infection under the conditions in which she was living; and the subsequent course of the trouble lent no support to the suspicion that she had contracted scarlatina. The pyrexia rapidly subsided; the rash speedily began to fade, and it was quite gone in four days; there was no sign of any peeling of the skin; and no one else in the neighbourhood developed scarlet fever.

The second case was that of a paralytic old man of sixty. The patient had hitherto been quite well in himself, except for sleeplessness and his paralysis. Each night he had been taking a seven-grain dose of veronal, and the day after the fifth of these he complained of dulness in the head and headache, with weary pains in his limbs, none of which symptoms had been complained of before. His temperature began to rise in the evening to 100° F. or thereabouts, and he broke out into a bright scarlatiniform rash over his chest, abdomen, and thighs. The veronal was stopped, and two days later the erythema had entirely gone again, and with it all the patient's unwonted symptoms.

These two cases agree exactly with those recorded by others as instances of veronal eruptions. It would seem as if certain patients have a peculiar idiosyncrasy to the drug; for in comparison to the very large numbers of persons who can take veronal well only a very small number show any of this tendency to erythema. It would have been a most interesting experiment to have tried veronal a second time in the above patients, to see whether the erythema would have recurred. It was scarcely justifiable to make the experiment, however, and they are by no means the only cases in which a scarlatiniform eruption after the administration of veronal has been recorded.

